# Altered Glycosylation Contributes to Placental Dysfunction Upon Early Disruption of the NK Cell-DC Dynamics

**DOI:** 10.3389/fimmu.2020.01316

**Published:** 2020-07-14

**Authors:** Sophia Borowski, Irene Tirado-Gonzalez, Nancy Freitag, Mariana G. Garcia, Gabriela Barrientos, Sandra M. Blois

**Affiliations:** ^1^Experimental and Clinical Research Center, A Cooperation Between the Max Delbrück Center for Molecular Medicine in the Helmholtz Association, and the Charité—Universitätsmedizin Berlin, AG GlycoImmunology, Berlin, Germany; ^2^Department of Obstetrics and Fetal Medicine, University Medical Center Hamburg-Eppendorf, Hamburg, Germany; ^3^Georg-Speyer-Haus, Institute for Tumor Biology and Experimental Therapy, Frankfurt, Germany; ^4^Instituto de Investigaciones en Medicina Traslacional, Facultad de Ciencias Biomédicas, CONICET, Universidad Austral, Derqui-Pilar, Argentina; ^5^Laboratorio de Medicina Experimental, Hospital Alemán—Consejo Nacional de Investigaciones Científicas y Técnicas, Buenos Aires, Argentina

**Keywords:** dendritic cells, natural killer cells, implantation, glycoimmunology, placentation

## Abstract

Immune cells [e. g., dendritic cells (DC) and natural killer (NK) cells] are critical players during the pre-placentation stage for successful mammalian pregnancy. Proper placental and fetal development relies on balanced DC-NK cell interactions regulating immune cell homing, maternal vascular expansion, and trophoblast functions. Previously, we showed that *in vivo* disruption of the uterine NK cell-DC balance interferes with the decidualization process, with subsequent impact on placental and fetal development leading to fetal growth restriction. Glycans are essential determinants of reproductive health and the glycocode expressed in a particular compartment (e.g., placenta) is highly dependent on the cell type and its developmental and pathological state. Here, we aimed to investigate the maternal and placental glycovariation during the pre- and post-placentation period associated with disruption of the NK cell-DC dynamics during early pregnancy. We observed that depletion of NK cells was associated with significant increases of O- and N-linked glycosylation and sialylation in the decidual vascular zone during the pre-placental period, followed by downregulation of core 1 and poly-LacNAc extended O-glycans and increased expression of branched N-glycans affecting mainly the placental giant cells and spongiotrophoblasts of the junctional zone. On the other hand, expansion of DC induced a milder increase of Tn antigen (truncated form of mucin-type O-glycans) and branched N-glycan expression in the vascular zone, with only modest changes in the glycosylation pattern during the post-placentation period. In both groups, this spatiotemporal variation in the glycosylation pattern of the implantation site was accompanied by corresponding changes in galectin-1 expression. Our results show that pre- and post- placentation implantation sites have a differential glycopattern upon disruption of the NK cell-DC dynamics, suggesting that immune imbalance early in gestation impacts placentation and fetal development by directly influencing the placental glycocode.

## Introduction

In hemochorial placentation, the placental trophoblasts have direct contact to maternal immune cells. Thus, trophoblast cells are exposed to allogenic immune responses by the mother. Uterine immune responses must be regulated in a way that allows access of the placenta to the maternal blood supply but also prevents excess invasion of fetal cells and infections (1). For successful pregnancy, maternal tolerance to the fetus needs to be established, otherwise failure of the maternal immune response to adapt correctly can lead to aberrant immune activation, which is associated with preeclampsia and miscarriage (2).

Highly specialized subpopulations of maternal leukocytes, such as uterine NK (uNK) cells, infiltrate the murine decidua in large numbers during the first half of pregnancy (3, 4). Through expression of different factors (e.g., VEGF and IFN-γ), uNK cells guide the remodeling of decidual spiral arteries increasing the availability of maternal blood at the implantation site and promoting trophoblast invasion (5–7). Another important subpopulation of maternal leukocytes key for modulation of local immunity and tolerance are uterine DC (uDC), which increase in number during the pre-placentation period, reaching a plateau in the post-placentation phase (8). These cells also support vascular adaptations during pregnancy including vessel permeability and blood flow to the implantation site through the CXCL12/CXCR4 pathway (9–11). Recruitment of NK cells, which is facilitated by DC, represents a mechanism to confine the immunogenic potential of uDC. Thus, healthy dynamics in the proportion of uNK cells and uDC during pregnancy play a critical role not only in the regulation of angiogenesis and decidualization (11, 12) but also in the placentation process. Immune cell imbalance during early pregnancy, such as expansion of DC or depletion of NK cells, has an effect on the pre-placentation period and also on the placental phenotype (13). For instance, implantation sites from NK cell depleted dams showed decidual growth defects during early pregnancy, indicated by a disrupted dynamics of decidua maturation (12). Additionally, these mice exhibited vascular defects (i.e., narrow lumens and cuffed appearance) in the central, proximal region of the decidua basalis during the post-placentation period together with increased accumulation of vascular- and tissue-associated NK cells in the mesometrial lymphoid aggregate of pregnancy (13). As a result from placental insufficiency, fetuses derived from NK cell depleted dams suffer from intrauterine growth restriction (IUGR) accompanied by an overall reduction of global DNA methylation levels and epigenetic changes in the methylation of specific hepatic gene promoters. Likewise, the expansion of DC during early pregnancy also provoked decidual growth defects on E5.5 (12) and changes in immune cell recruitment, with increased numbers of perivascular DC at the mesometrial decidua (MD) (11) and upregulation of IL-10 expressing NK cells on E7.5 (14). Expansion of DC also led to significant changes in placental morphology, with impaired vascular development of the labyrinth and an increased accumulation of glycogen cells in the junctional zone (13), but the effect on pregnancy outcome was milder as offspring derived from these pregnancies did not suffer from IUGR and exhibited slight gene-specific epigenetic changes.

**Figure 1:**
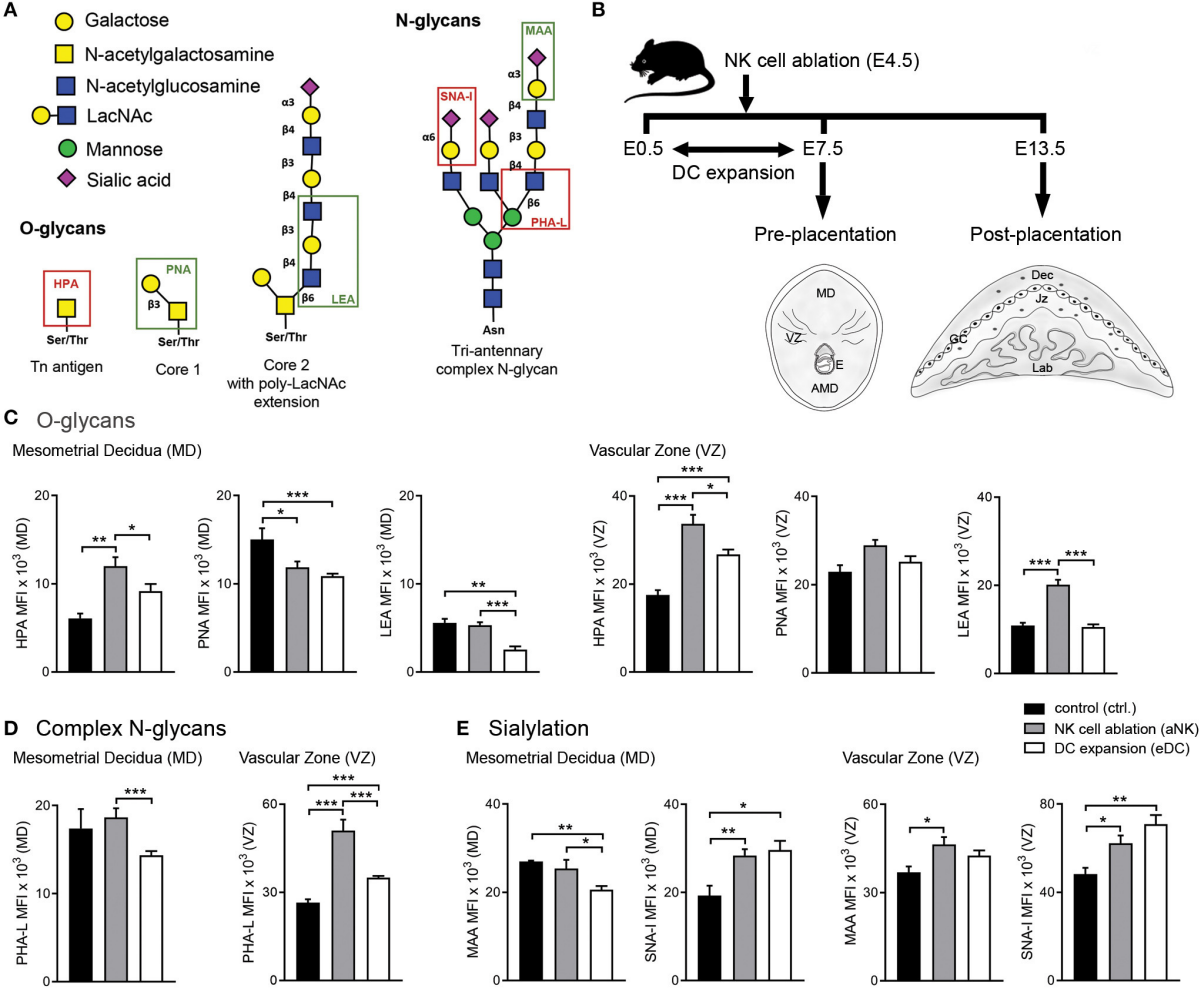
Influence of NK cell depletion and DC expansion on the glycophenotype of implantation sites during the pre-placentation period. **(A)** For analysis of the glycophenotype, lectins were used to detect different types of glycosylation. O-glycan structures were recognized by Helix pomatia agglutinin (HPA; Tn-antigen), Arachis hypogaea lectin (PNA; core 1), and *L*ycopersicon esculentum lectin (LEA; core 2). In addition, we employed *Phaseolus vulgaris* lectin (PHA-L), which specifically recognizes β1-6GlcNAc-branched complex N-glycans. Finally, sialyation was determined using the Maackia amurensis lectin (MAA) and Sambucus nigra agglutinin (SNA-I) which bind to α2,3- and α2,6-linked sialic acid, respectively. **(B)** Experimental design: pregnant CD11c.DTR females were injected (i.p.) on E4.5 with anti asialo GM1 for transient ablation of NK cells. For the expansion of uterine DC, pregnant CD11c.DTR females were treated with one daily injection of FL (10 mg/mouse/day) from E0.5 to E7.5 as described in material and methods. Pre- (E7.5) and post-placentation (E13.5) period implantation sites were included in the glycodynamics analysis. **(C–E)** Quantification of O-glycan **(C)**, complex N-glycan **(D)**, and sialylated glycan **(E)** mean fluorescence intensity (MFI) in the mesometrial decidual (MD), and vascular zone (VZ) of implantation sites following NK cell ablation or DC expansion during the pre-placentation stage. In all figures, data shown are mean ± S.E.M. and differences are denoted as *P < 0.05, **P < 0.01, and ***P < 0.001, as analyzed by *Mann-Whitney U-test*. AMD, antimesometrial decidua; Dec, decidua; MD, mesometrial decidua; VZ, vascular zone; GC, giant cells; Jz, junctional zone; Lab, labyrinth.

Glycans are sequences of carbohydrates that are added to proteins and lipids to modulate their structure and function (15). Two major types of glycosylation are observed: N-linked glycosylation is the attachment of oligosaccharides to asparagine or arginine side-chains, whereas O-linked glycosylation occurs mainly at serine and threonine (Figure 1A). Glycans modify proteins required for trophoblast function, and alterations have been associated with pathological conditions. Thus, aberrant N-glycosylation of integrin β1 in villous tissues, which influences trophoblast invasion, was linked to early spontaneous miscarriage in humans (16). Lectin histochemistry analyses performed in human placentas revealed significant alterations of carbohydrate metabolism (i.e., dysregulation of α-D-mannose, GlcNAc, β-GalNAc, and α-Fucose) after the onset of different types of hypertensive disorders and fetal growth restriction (17, 18); showing for instance alterations in the trophoblast and/or endothelial cell glycophenotype of the pathological groups (17) and an altered distribution of α2,3 and α2,6-linked sialic acid in placentas from hypertensive disorders (18). More recently, Tannetta et al. showed that preeclampsia is associated with changes in the surface glycosylation of syncytiotrophoblast derived extracellular vesicles (STBEVs), which are released in increased numbers and exhibit a proinflammatory, anti-angiogenic, and procoagulant activity. Indeed, STBEVs derived from preeclamptic patients exhibited increased binding of *Sambucus nigra* lectin and *Ricinus communis* agglutinin I, which bind to α2,6-linked sialic acid and galactose or N-acetylgalactosamine residues (19), which may be a link to changes in vesicle-cell interactions affecting functions like cell targeting, clearance, and immune activity. However, further investigation is needed to determine whether and how different alterations in glycosylation contribute to inappropriate maternal-fetal immune responses and poor pregnancy outcomes. In this work, we analyzed the effect of temporary changes within the DC or NK cell pool during early pregnancy on the glycophenotype during the pre- and post-placentation process, before the onset of the IUGR disease phenotype. We show that pre- and post- placentation implantations have a differential glycopattern where either NK cells were temporally ablated or DC were expanded. Our data confirm that immune dysregulations early in gestation have an impact on the placental glycocode, influencing the placentation process itself and subsequently fetal development.

## Materials and Methods

### Animals

All animals tissues used in this work were collected for previous experiments assessing the role of NK cell—DC interactions in the modulation of early pregnancy maternal adaptations, placentation and fetal growth (11–13) in accordance with guidelines for the care and use of laboratory research animals promulgated by the Charité—Universitätsmedizin Berlin and Regional Office for Health and Social Affairs. Animals were purchased from Jaxmice® and maintained on a 12L/12D cycle. Five- to six-weeks old CD11c.DTR females were mated with Balb/c males. The presence of a vaginal plug after cohabitation was denoted as embryonic day (E) 0.5. Females were kept in groups of 4–5 animals and injected (i.p.) on E4.5 with anti asialo GM1 (WAKO, Cat.no. 986-10001, 2 mg/g BW) for transient ablation of NK cells (aNK group, Figure 1B). For the expansion of uterine DC (eDC group, Figure 1B), Balb/c-mated CD11c.DTR females were treated with one daily injection of human recombinant Fms-related tyrosine kinase 3 ligand (FL; BioX cell, Cat.no. BE0098, 10 mg/mouse/day) from E0.5 to E7.5. Control CD11c.DTR females received PBS supplemented with rabbit normal serum (2 mg/g body weight i.p.). On E7.5 and 13.5, mice from the respective groups were sacrificed and uterine tissue from the implantation sites (*n* = 5) was processed for histological sectioning according to standard procedures. Pregnancy outcomes for the different groups have been described in our previous studies (11–13).

### Immunofluorescence

We used a panel of lectins that recognize specific O-glycan structures (Helix pomatia agglutinin (HPA; Tn-antigen), Arachis hypogaea lectin (PNA; core 1), and *L*ycopersicon esculentum lectin [LEA; core 2)]. In addition, we employed *Phaseolus vulgaris* lectin (PHA-L), which specifically recognizes β1,6GlcNAc-branched complex N-glycans. Finally, sialyation was determined using the Maackia amurensis lectin (MAA) and Sambucus nigra agglutinin (SNA-I) which bind to α2,3- and α2,6-linked sialic acid, respectively (Figure 1A). Serial cryosections of implantation sites were prepared at 8 μm. Briefly, slides were washed in TBS and blocked with Biotin Blocking system (X0590, DAKO Corporation) for 20 min in a humid chamber at RT. Afterwards, slides were blocked with Carbo-Free Blocking Solution (SP-5040, Vector Laboratories) for 30 min in a humid chamber at RT. Subsequently, slides were incubated with biotinylated lectin (EY Laboratories) diluted in Carbo-Free Blocking Solution for 16 h at 4°C in a humid chamber HPA (20 ng/ml; BA-3601-1), PHA-L (20 ng/ml; BA-1801-2), or SNA-I (10 ng/ml; BA-6802-1). Lectin-stained sections were then incubated with 2 μg/ml Streptavidin-Tetramethylrhodamine (S-870; Invitrogen) for 1 h in a humid chamber at RT. Subsequently, slides were incubated with FITC-labeled lectin (EY Laboratories) diluted in Carbo-Free Blocking Solution for 2 h at RT PNA (20 ng/ml; F-2301-1), LEA (20 ng/ml; F-7001-1), or MAA (20 ng/ml; F-7801-2). Nuclei were counterstained with 4′,6-diamidino-2-phenylindole (DAPI) for 5 min at RT and mounted in Prolong Gold (P36930, Invitrogen). Stainings of whole implantation sites were digitally scanned by a high-resolution bright field and fluorescence slide scanner (Pannoramic MIDI BF/FL, 3DHISTECH Ltd.), and staining was evaluated on virtual slides using Pannoramic Viewer 1.15.4 (3DHISTECH Ltd.) by two examiners blinded to the experimental group.

### Galectin-1 Staining

Staining of 8 μm cryo sections was performed by washing in TBS, followed by blocking with Duale Endogenous Enzyme Block (S2003, Dako) for 30 min in a humid chamber at RT. Afterwards, slides were blocked with Proteinblock (PHA-70873, Dianova) for 20 min. The primary antibody against galectin-1 (1:400; GTX 101566, GeneTex) was incubated over night at 4°C. The slides were than washed and incubated with HRP-conjugated secondary antibody (111-035-003; Jackson ImmunoResearch) for 1 h at RT. The signal was detected by incubation at RT with a 0.05% diaminobenzidine in 0.015% H_2_O_2_ substrate solution. After washing, nuclei were counterstained with 0.1% Mayer's hematoxylin followed by a standard dehydration procedure and mounting in Entellan (Merck Millipore).

### Statistics

Data analysis was performed with GraphPad Prism 7 (GraphPad Software, Inc.). Data are presented as mean ± SEM and were analyzed with D'Agostino-Pearson omnibus normality test followed by unpaired *t*-test or Mann-Whitney test. A *p* < 0.05 was considered as significant.

## Results

### Dysregulation of the NK Cell or DC Pool Changed the Distribution of O-Glycans, Complex N-Glycans, and Sialylation in the Mesometrial Decidua and Vascular Zone During the Pre-placentation Period

In order to determine whether temporary ablation of NK cell or expansion of DC during early pregnancy could influence the glycophenotype of the implantation sites, we analyzed implantation sites during the pre-placentation period (on E7.5) focusing on the quantification within the mesometrial decidua (MD) and vascular zone (VZ) (Figure 1B).

We first examined the O-glycans during the pre-placentation period (Figure 1C). During normal gestation abundant expression of core 1 O-glycans (PNA) compared to Tn antigen (HPA) and core 2 O-glycans (LEA) in the MD was observed (Figure 1C, left panel). Depletion of NK cells during early pregnancy caused a decrease in core 1 O-glycans (PNA) and an increase of Tn antigens (HPA) in this region. In contrast, the expansion of DC during the pre-placentation period caused a slight increase in Tn antigens (HPA) and decreased expression of core 1 (PNA) and core 2 O-glycans (LEA). Under normal conditions, HPA reactive O-glycans were observed in the VZ on E7.5. Of note, HPA-reactivity was significantly increased in the VZ of the aNK and the eDC group compared to the control group, with the aNK group showing the highest MFI. No changes were observed in PNA reactive glycans. LEA staining was increased in the VZ of the aNK group but not in the eDC group compared to the control group (Figure 1C, right panel). Next, we examined the distribution of complex branched N-glycans (specifically MGAT5-modified) during the pre-placentation period (Figure 1D). Glycans bound by PHA-L were observed in the MD of all groups (Figure 1D, left panel), with comparable mean fluorescence intensities (MFIs) of the control and the aNK group. Notably, a significantly lower MFI in the MD of the eDC group was observed compared to the aNK group. Regarding the distribution of complex branched N-glycans within the VZ, binding of PHA-L showed that staining intensity was significantly increased in the aNK and the eDC group compared to the control group (Figure 1D, right panel). As for the distribution pattern of sialylated glycans in the MD, the control group and the aNK group showed comparable MFIs but the eDC group displayed a lower MAA MFI (Figure 1E, left panel). For SNA-I reactive glycans, similar MFIs in the aNK and the eDC group were observed at the MD during the pre-placentation period. Staining intensity for α2,3-linked sialic acid (MAA) was significantly increased in the VZ of the aNK group compared to the control group, whereas SNA-I reactive glycans showed a significant increase in the aNK and eDC dams (Figure 1E, right panel).

### Imbalance on NK or DC Cell Subsets During Early Gestation Provokes Altered O- and N-Glycosylation Patterns in the Post-placentation Period

**Figure 2:**
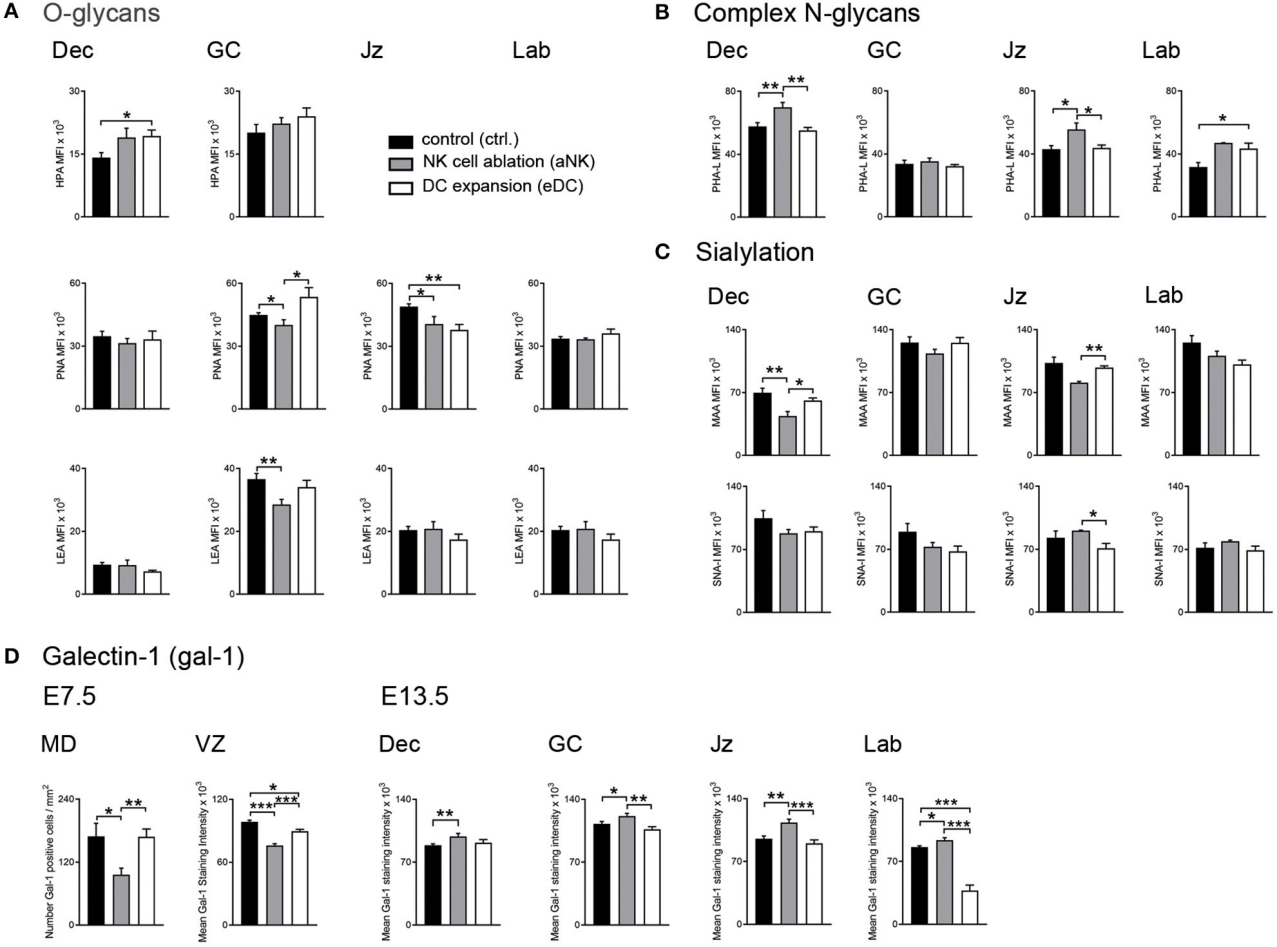
Placental glycocode dynamics upon NK cell depletion and DC expansion during early gestation. **(A)** Quantification of O-glycan distribution across decidua and placenta layers on E13.5. Tn antigen was identified using the Helix pomatia agglutinin (HPA), core 1 and core 2 O-glycans were detected by Arachis hypogaea lectin (PNA) and *L*ycopersicon esculentum lectin (LEA), respectively. **(B)** Expression patterns of complex N-glycans were detected by *Phaseolus vulgaris* lectin (PHA-L) on E13.5. **(C)** Sialylation in the post-placentation period was characterized using Maackia amurensis lectin (MAA) and Sambucus nigra agglutinin (SNA-I) which bind to α2,3- and α2,6-linked sialic acid, respectively. **(D)** Analysis of galectin-1 (gal-1) expression during the pre- (E7.5) and post-placentation (E13.5) period. In all panels, bars show the MFI mean values and the corresponding S.E.M. Differences are noted as *P < 0.05, **P < 0.01, and ***P < 0.001 according to *Mann-Whitney U-test*. MFI, mean fluorescence intensity; MD, mesometrial decidua; VZ, vascular zone; GC, giant cells; Jz, junctional zone; Lab, labyrinth.

Taking into account that alterations of NK cell and DC relative abundance were shown to influence the placentation process and epigenetic programming in the offspring (13), we next examined changes in the glycophenotype during the post-placentation period (E13.5). Figure 2A (upper panel) shows the distribution of O-glycans within the decidua and placenta. During normal gestation Tn antigen (HPA) was only observable in the decidua and on giant cells (GC). In contrast, core 1 (PNA, middle panels), and core 2 O-glycans (LEA, bottom panels) were observed in all layers of the implantation site (including decidua, GC, junctional zone (Jz), and labyrinth). More Tn antigen (HPA) was observed on GC trophoblast than in the decidua. Core 1 O-glycans (PNA) were abundantly expressed in all layers but core 2 O-glycans (LEA) were more abundant on GC than in the decidua. Depletion of NK cells during early pregnancy was associated with decreased levels of core 1 O-glycans (PNA) on GC and Jz and reduced expression of core 2 O-glycans (LEA) on GC (Figure 2A, middle and bottom panels). In contrast, expansion of DC provoked an increase of Tn antigen (HPA) in the decidua (Figure 2A, upper panel), accompanied by increased expression of core 1 O-glycans (PNA) on GC but reduced expression in the Jz (middle panels). When analyzing the complex branched N-glycans (PHA-L, Figure 2B), we observed that during the post-placentation period reactivity in the decidua is stronger than in the placenta in undisturbed pregnancy. Expression of branched, complex N-glycans (PHA-L) was increased in the decidua and the Jz of the aNK group, but only in the labyrinth of eDC placentas. Finally, analysis of sialyation showed that MAA-reactive α2,3-linked sialic acid was detected on giant cells and in the labyrinth under normal placentation (Figure 2C, upper panel), accompanied with a strong expression of α2,6-linked sialic acid (SNA-I, bottom panel) in the decidua. Compared to controls, depletion of NK cells during early pregnancy provoked a decrease of α2,3-sialylation in the decidua and the Jz and an increase of α2,6-sialylation in the Jz, whereas placentas derived from DC expanded dams showed a decrease of α2,6-sialylation in the Jz.

### Alteration of the Glycosylation Signature During the Pre- and Post-placentation Period is Accompanied by Changes on Gal-1 Expression

Given its well-established role in the modulation of pregnancy associated processes (20, 21), our next aim was to characterize galectin-1 (gal-1) expression during the pre- (E7.5) and post- (E13.5) placentation period. During the pre-placental period, we observed reduced gal-1 expression on the mesometrial decidua upon NK cell depletion compared to untreated dams (Figure 2D, left panel), whereas MD expression of this lectin was not sensitive to DC expansion. In contrast, both treated groups (aNK and eDC) exhibited decreased levels of gal-1 expression in the VZ, especially on endothelial cells during the pre-placentation period. As pregnancy progressed to the post-placentation period, aNK dams showed increased gal-1 expression within the decidua and placental layers (including GC, Jz, and labyrinth) compared to controls (Figure 2D, right panel). However, eDC placentas showed decreased gal-1 levels on the GC and the labyrinth on E13.5, suggesting that changes of gal-1 expression together with an altered glycosylation signature could interfere with the pregnancy protective functions of this lectin.

## Discussion

**Figure 3:**
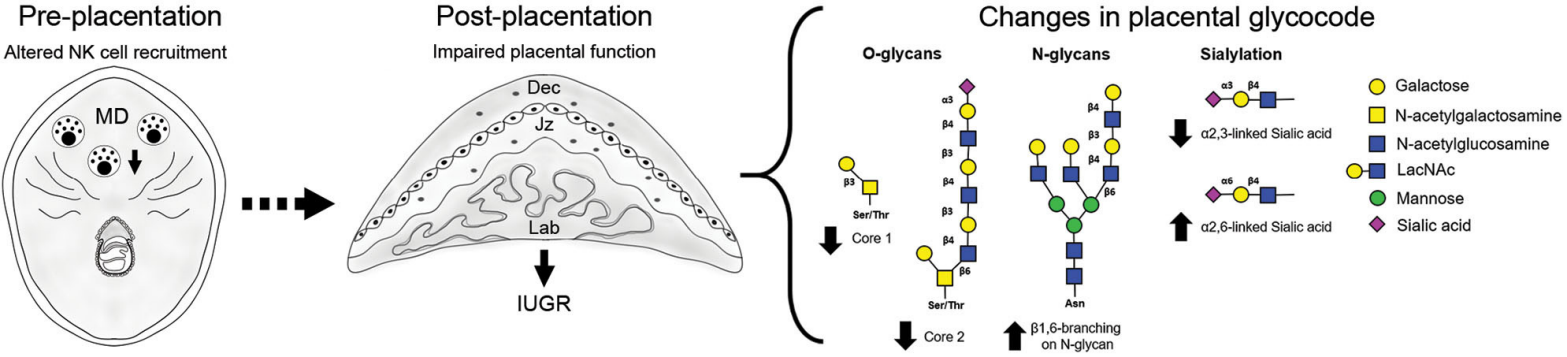
Overview of the placenta glycocode dynamics in poor pregnancy outcome caused by disrupted NK cell recruitment. Fetal growth restriction as a consequence of NK cell depletion is associated with changes in O-glycan expression (↓ core 1 and core 2) in giant cells (GC). Junctional zone is characterized by increased expression of branched N-glycans and changes in sialylation (↓ α 2, 3- and ↑ α 2, 6- linked sialic acid). Placentas derived from NK ablated dams are characterized by an increased gal-1 expression.

Changes in local immune cells dynamics (e.g., uNK cells and DC) during early gestation lead to the development of placental abnormalities and particularly upon NK cell depletion, fetal growth restriction (11–13). Our study on gal-1-glycan circuits in mice shows that changes in immune cell subset frequencies during the pre-placentation period differentially alter the placental glycophenotypes: placenta derived from NK depleted dams displayed reduced expression of O-glycans and α2,3-sialylation in placental layers accompanied by upregulation of complex N-glycans (Figure 3). This does not seem to be the case in placenta derived from expanded DC dams, which by contrast displayed milder changes in the placental glycophenotype with a modest reduction of core 1 O-glycosylation and α2,6-sialylation specially in the junctional zone and only a slight increase of N-glycans in the labyrinth.

Our study has limitations regarding the challenges of studying diversity on glycopatterns and the lack of *in vitro* experimentation, with specific consideration for the technical difficulty to preserve glycan structure and mimic the complex glycovariations in an *in vitro* setting. Nevertheless, the results reported herein highlight the notion that balanced innate immune cell dynamics at the maternal fetal interface have a strong impact on the glycophenotype, thereby influencing galectin-glycan interactions driving decidual and placental functions.

### Pre-placentation Impaired NK Cell-DC Dynamic Alters Glycopatterns Within the Maternal Vascular Decidua

During early gestation, NK cells and DC shape decidual adaption to the developing embryo regulating angiogenesis and vascular growth (11–13). We have previously shown that DC found associated with the decidual vasculature co-express CXCR4 and impaired homing of CXCR4^+^DC altered decidual vascular organization with impaired spiral artery remodeling later in gestation (11). In this study, we further reveal that alterations on the NK cell and DC pool dynamics during the pre-placentation period affect the glycopattern of the vasculature at the feto-maternal interface. In this regard, the VEGF system plays a paramount role in uterine vascular permeability and angiogenesis during implantation and decidualization (22, 23) and several findings have highlighted the importance of glycosylation for VEGFR2 functionality. For instance, VEGF-dependent proliferation is influenced by heparan sulfate (24) and complex branched N-glycans on the VEGFR2 are responsible for gal-1/VEGF-like signaling to sustain angiogenesis (25). Sialylation on VEGFR2 can also determine the signaling capacity of this receptor through gal-1. Thus, α2,6- linked, but not α2,3-terminal sialic acid inhibits binding of gal-1, which can also bind to the VEGFR2 to activate alternative pro-angiogenic signaling (25, 26). Additionally, exposure of endothelial cells to hypoxic conditions leads to increased branching of β1,6 branched N-glycan structures, and elongation of poly-LacNAc residues on core 2 O-glycans (25). These examples highlight the versatility of the endothelial glycome and its ability to adapt to cellular physiology. Indeed, several of these changes in the glycosylation pattern of the vascular zone during the pre-placentation period were observed in the present study upon DC expansion or NK cell ablation. Ablation of NK cells provoked an increase of core 2 O-glycans, branched N-glycans, and α2,3-sialyation compared to the control group, indicating the possibility of hypoxic or inflammatory conditions and increased gal-1 binding. These changes may occur to compensate the low gal-1 levels due to reduced NK cell abundance in these implantation sites. Expansion of DC, on the other hand, led to increased expression of branched N-glycans and α2,6-linked sialic acid compared to the control group; which despite not affecting the normal VEGF/VEGFR2 signaling pathway may lead to lower gal-1 sensitivity of cells in the vascular zone of this group. The corollary to these observations is that the decidual vascular glycocode appears to be dependent on the concerted actions of NK cells and DC, by virtue of their effect as modulators of VEGF/ gal-1 signaling pathways.

Thickness of the glycocalyx covering endothelial cells can influence the access of leukocytes to adhesion receptors on the endothelial cell surface. Pro-inflammatory cytokines, such as TNF-α, can lead to disruption of the endothelial glycocalyx and thus to an increase in leukocyte recruitment (27, 28). In this context, immune cell imbalance (i.e., DC expansion or NK cell depletion) during early pregnancy may influence the cytokine profile at the implantation site, leading to altered properties of the endothelial glycocalyx by directly influencing the expression of glycosyltransferases. Indeed, our previous studies have shown that expansion of DC was associated with a significant upregulation of the CXCL12/CXCR4 pathway; which has recently been shown to enhance megakaryocyte expression of *B4GalT1* (29), one of the main galactosyltransferases involved in the synthesis of the LacNAc moieties present in core 2 O-glycans and complex N-glycans. In turn, since B4GalT1-dependent galactosylation modulates β1 integrin function (29), such cytokine-mediated changes in the endothelial glycocalyx may further contribute to immune disbalance by provoking a differential recruitment of leukocytes due to altered cell adhesion properties. Indeed, DC expansion or NK cell depletion induced several changes in the glycosylation pattern in the vascular zone during the pre-placentation period, particularly in the expression of Tn antigen. In addition, endothelial gal-1 has been shown to reduce lymphocyte recruitment (30), further indicating that in the aNK group, which showed reduced gal-1 staining of endothelial cells, lymphocyte trafficking might be enhanced compared to the control group.

### Pre-placentation Manipulation of the Relative NK Cell-DC Abundance Modifies Gal-1 Binding Placental Glycophenotypes

Trophoblast glycodiversity is part of the trophoblast lineage identity (31). Several pregnancy complications including preeclampsia, IUGR, and miscarriages were associated with specific differential glycosylation patterns after the onset of the disease (16–19, 32). In a first effort to identify early glycosignals that influence placental development upon disruption of the NK cell-DC dynamics, we show here that changes in trophoblast glycosylation patterns precede poor pregnancy outcomes (e.g., IUGR). For instance, Tn antigen O-glycans are exclusively expressed on the giant cell layer of the placenta and to a lesser extent in the decidua during unchallenged pregnancy. Both depletion of NK cells or expansion of DC in absence of dangers signals increased Tn antigen expression in the decidua. Since Tn antigen expression has been linked to enhanced growth and invasion ability in cancer cells (33–35), it is possible that increased decidual Tn antigen expression would act to facilitate trophoblast invasion. In this regard, trophoblast giant cells showed intense staining with LEA, indicating increased expression of LacNAc core 2 O-glycans during normal pregnancy. Giant cells in particular need to acquire an invasive character to make contact to the maternal arteries and replace the endothelial cell lining of the maternal blood vessels to funnel blood into the placenta. Importantly, our results further showed a down-regulation of core 2 O-glycans on giant cells derived from NK ablated dams. As cell surface mucin 1 (MUC1) carrying core 2 O-glycans is involved in trophoblast migration and adhesion to uterine endothelial cells (36–39), data suggests that changes in MUC1 core 2 O-glycans pattern would interfere with the invasive properties of giant cells in NK ablated placentas. This is in agreement with our previous work showing that aNK mice had impaired spiral artery remodeling and IUGR (13), indicating that a differential glycosylation pattern in the post-placentation period results in poor spiral artery remodeling. Moreover, expression of core 1 O-glycans has also been detected on MUC1 in the human placenta (40). In our study, staining of core 1 O-glycans by PNA also revealed reduced expression on trophoblast giant cells (aNK group) and trophoblasts in the junctional zone (aNK and eDC group), which could further indicate alterations in mucin expression or glycosylation. Considering that gal-1 is able to bind mucins on trophoblast cells and is involved in the trophoblast invasion machinery (41, 42), the increased gal-1 expression in aNK placenta may represent an attempt to compensate reduced abundance of MUC1 binding partners.

Enhanced expression of N-acetylglucosaminyl transferase V (GnTV) characterizes first trimester placentas in normal gestation (43). GnTV generates β1-6-N-acetylglucosamine branches in complex N-glycans, which are recognized by gal-1. In this context, LacNAc motives are a glycan signature of invasive trophoblast cells not only on their surface but also on their secretion product HLA-G (31, 44, 51). The significantly higher expression of complex, branched N-glycans detected in the junctional zone of the aNK group indicates that the middle connecting layer of the placenta efficiently glycoadapts to the maternal environment giving rise to trophoblast giant cells and glycogen cells that invade and anchor the placenta to the decidua (45). In addition, we observed a switch on sialylation from α 2,3-linked to α2,6-linked sialic acid in the labyrinth of the aNK group. This finding correlates with the reduced fetal vascular density in the labyrinth upon NK depletion and with the inflammatory status due to the increased NK cell density in the mesometrial lymphoid aggregate of pregnancy (13). Interestingly, changes in the glycosylation status predominantly affecting the placental labyrinth and junctional zone have been reported in a rat model of hyperglycemic placental dysfunction (46, 47); suggesting that glycovariations in these layers induced by adverse maternal environments may have direct impact on placental function.

Our results further showed that increased α2,6 sialylation can reduce gal-1 mediated angiogenesis (48), which is critical for healthy placentation (49). Moreover, the inhibition of gal-1 binding by sialylation at the position six of galactose has been suggested to make T cells resistant to apoptosis (50) and might contribute to uncontrolled maternal inflammation during pregnancy complications (20, 49). Indeed, increased α2,6 sialylation in STBEV surface has been associated with human PE syndrome (19). Taken together, the results reported here highlight the relevance of glycodynamics during the pre- and post-placentation period that could be helpful to the understanding of the pathogenesis of poor pregnancy outcomes.

## Data Availability Statement

The raw data supporting the conclusions of this article will be made available by the authors, without undue reservation.

## Ethics Statement

The animal study was reviewed and approved by Charité—Universitätsmedizin Berlin and Regional Office for Health and Social Affairs.

## Author Contributions

SMB designed the study and secured grant funding. SB, IT-G, GB, NF, SMB, and MG performed experiments and/or analyzed data. SB, GB, and SMB wrote the manuscript. All authors gave approval for publication.

## Conflict of Interest

The authors declare that the research was conducted in the absence of any commercial or financial relationships that could be construed as a potential conflict of interest.
